# Perioperative Antimicrobial Prophylaxis for Invasive Dental Procedures: A Systematic Review and Random-Effects Meta-Analysis of Randomized and Placebo-Controlled Studies

**DOI:** 10.7759/cureus.100755

**Published:** 2026-01-04

**Authors:** Alberto A Iturbe Cordero, Arturo P Jaramillo, Jhanmarie Vasquez

**Affiliations:** 1 General Dentistry, Universidad Central de Venezuela, Caracas, VEN; 2 General Practice, Universidad Estatal de Guayaquil, Guayaquil, ECU; 3 Dentistry, University North Oriental Grand Marshal of Ayacucho, Barcelona, VEN

**Keywords:** dental material science, dental procedures, dental surgery, oral implants, oral prophylaxis

## Abstract

Prophylactic antimicrobials are frequently administered before invasive dental procedures (including implant placement and extractions) to reduce early infectious complications and procedure-related bacteremia; however, clinical benefit remains debated and must be balanced against ecological disruption and antimicrobial resistance. Evidence from experimental work indicates that even a single prophylactic dose of amoxicillin may transiently perturb the oral microbiome and select resistant strains.* *A Preferred Reporting Items for Systematic Reviews and Meta-Analyses (PRISMA)-guided systematic review was conducted. PubMed, Embase, and Cochrane databases were searched. *S*creening identified 10 randomized placebo-controlled/comparator trials (486 records; 289 duplicates removed; 197 screened; 72 full texts reviewed) for qualitative synthesis and meta-analysis. Included studies evaluated perioperative prophylaxis (systemic antibiotics and/or local antiseptic prophylaxis) versus placebo or no prophylaxis in invasive dental procedures. Outcomes were harmonized across studies as early procedure-related infectious outcomes and/or bacteremia-related endpoints, with secondary assessment of postoperative morbidity indicators (e.g., pain/analgesic use where available). Meta-analysis was performed in Review Manager (RevMan) version 5.4 (2020; The Cochrane Collaboration, London, United Kingdom), using an inverse-variance random-effects model.

Across all invasive dental procedures (~1,950 participants pooled), prophylaxis did not demonstrate a statistically significant overall reduction in the primary pooled endpoint (MD −0.12, 95%CI −0.31 to 0.07; random-effects; I² ≈ 99%). In the dental-surgery subgroup, the pooled effect similarly crossed the null (MD −0.17, 95%CI −0.45 to 0.11; I² ≈ 99%). Individual trials showed marked reductions in post-extraction bacteremia surrogates with chlorhexidine mouthwash prophylaxis and with intravenous amoxicillin-clavulanate in extraction settings, whereas in uncomplicated extractions among well-controlled type 2 diabetes, antibiotics did not reduce postoperative complications and were associated with greater analgesic consumption. Implant trials reported low event rates and did not consistently demonstrate clinically meaningful superiority of routine prophylaxis. Funnel plot inspection suggested possible small-study effects influenced by outliers, but interpretation was constrained by extreme between-study heterogeneity.* *In this pooled analysis of randomized and placebo-controlled studies spanning heterogeneous invasive dental procedures, routine prophylactic antimicrobial strategies did not yield a consistent overall benefit on early infectious/bacteremia-related outcomes, and secondary postoperative morbidity outcomes showed no clear improvement. In parallel, microbiological evidence indicates that single-dose prophylaxis can promote short-term ecological disturbance and selection of resistant oral flora. Future trials should standardize endpoints (distinguishing clinical infection/implant failure from surrogate bacteremia measures), stratify by baseline risk, and prioritize antimicrobial stewardship to identify the limited patient subsets most likely to benefit.

## Introduction and background

Invasive dental procedures, particularly dental implant placement and tooth extraction, are among the most frequently performed interventions in oral healthcare and are increasingly delivered in outpatient settings. Although these procedures are generally safe, their sheer volume means that even low event rates translate into substantial absolute numbers of postoperative complications and unplanned follow-up visits [1-5]. Clinically, the outcomes that matter most to patients and providers include early infection, delayed healing, alveolar osteitis, postoperative pain and swelling, and, within implant therapy, early implant failure [4-9]. Because these adverse events can carry functional, financial, and psychosocial consequences, perioperative strategies intended to prevent early complications have received sustained attention, with systemic antibiotic prophylaxis often positioned as the most direct and easily implementable option. However, routine antibiotic use in low-risk dental surgery has become controversial, largely because the expected benefit may be modest while the public health costs of unnecessary exposure, particularly antimicrobial resistance, are cumulative and increasingly visible [7-10].

The rationale for antibiotic prophylaxis in dentistry rests on two main arguments. First, surgical manipulation of the oral cavity introduces bacteria into traumatized tissues, theoretically increasing the risk of postoperative infection and impaired wound healing. Second, dental procedures can induce transient bacteraemia, a phenomenon that has historically been linked to concerns about systemic sequelae in susceptible individuals and has driven the use of perioperative antibiotics in certain contexts. Trials within extraction settings illustrate how this debate extends beyond local outcomes. A randomized clinical trial (RCT) evaluating chlorhexidine prophylaxis in tooth extraction demonstrated measurable effects on post-extraction bacteremia [1], supporting the concept that procedural measures can influence bloodstream bacterial dissemination [11]. Similarly, an RCT testing intravenous amoxicillin-clavulanate during dental procedures was explicitly designed around bacteraemia prevention [3], reflecting the persistent clinical focus on bacteremia-related endpoints even as broader antibiotic stewardship pressures intensify [2,12].

At the same time, implant dentistry raises a distinct but related concern: the prevention of early implant loss. Early implant failure, typically occurring before prosthetic loading, represents a particularly consequential endpoint because it signals loss of osseointegration, interrupts treatment plans, and may necessitate additional surgery. As implant utilization expands and patient complexity increases, clinicians face ongoing uncertainty about whether a single preoperative antibiotic dose meaningfully reduces early failures [4,13,14]. Large, multicenter randomized evidence has been developed to address this question. A placebo-controlled double-blind trial conducted across multiple clinics evaluated a preoperative 2 g amoxicillin regimen compared with placebo and reported implant failures in both groups, underscoring that early failure can occur despite prophylaxis, and that baseline risk is often low in broadly healthy populations [5,6,13]. Additional randomized implant trials have evaluated similar prophylactic approaches, including single-dose regimens designed for pragmatic implementation, while tracking early failure and postoperative complications as key endpoints [6,7,9]. In parallel, a placebo-controlled trial evaluating preoperative clindamycin sought to determine whether an alternative antibiotic strategy could reduce early oral implant failure, reflecting real-world prescribing patterns in patients with penicillin allergy labels or clinician preference for non-beta-lactam options [7].

Tooth extraction provides an equally important clinical model for prophylaxis debates because it spans a wide spectrum of complexity, from routine simple extraction to surgical third-molar removal, and includes patient groups with potentially elevated risk [15-20]. A randomized multicenter clinical trial examining amoxicillin in simple dental extractions highlights the continued uncertainty around “routine” prophylaxis in everyday practice, especially when postoperative infection rates are already low, and the magnitude of absolute benefit is difficult to detect [6]. Surgical third-molar removal, often accompanied by swelling and trismus, has also been studied using rigorous designs; for example, a randomized, double-blind, placebo-controlled split-mouth trial assessed whether perioperative amoxicillin reduces surgical site infection and postoperative morbidity after routine removal of non-inflamed third molars [5]. Special populations introduce additional complexity: in controlled type 2 diabetes, a randomized trial comparing amoxicillin to placebo for single uncomplicated extractions evaluated perceived recovery and clinical healing endpoints, reflecting clinician concern that metabolic disease might heighten infectious risk or impair healing even in otherwise straightforward procedures [6].

Importantly, the modern assessment of prophylaxis must account for biological trade-offs that are not captured by short-term clinical complication counts. Even a single antibiotic dose can exert selective pressure on the oral microbiota [4,7,9,12]. Experimental evidence in healthy volunteers given a single 2 g dose of amoxicillin demonstrated ecological disturbance within oral flora and a measurable rise in reduced susceptibility among commensal streptococci, reinforcing the plausibility that “one-off” prophylaxis is not microbiologically neutral [2]. This is highly relevant for dentistry, where prophylactic exposures can be widespread and repeated across a population, potentially amplifying selection pressure at scale. Thus, the clinical question is no longer simply whether prophylaxis can reduce an adverse outcome, but whether any achievable benefit is sufficiently consistent and sufficiently large to justify predictable ecological consequences [8,16,18].

Despite multiple RCTs and procedure-specific studies, uncertainty persists because the evidence base is heterogeneous by design. Studies vary in procedure type (simple extraction, third-molar surgery, implant placement), prophylactic strategy (systemic antibiotics vs procedural antisepsis), population risk profile (healthy participants vs metabolic comorbidity), and outcome selection (clinical infection, early implant failure, symptom-based recovery metrics, or bacteremia-related endpoints) [1,3-7,9,10,15,20]. This diversity is reflected in pooled analyses derived from the provided forest plots: across invasive dental procedures, the combined effect estimate did not show a clear overall advantage of prophylaxis and was accompanied by marked inconsistency between studies, indicating that any “average” effect may conceal meaningful context-specific differences (e.g., procedure complexity or baseline risk) [1,3-10]. Furthermore, the presence of a contemporary synthesis focused on implant surgery, aimed specifically at early implant failure, illustrates that the question remains active and that conclusions may shift depending on inclusion criteria, endpoint prioritization, and risk-of-bias considerations [8,17].

Accordingly, the purpose of the present systematic review and meta-analysis was to identify, analyze, and integrate the comparative evidence, spanning implant surgery, extraction-based dental surgery, and mechanistic microbiological outcomes. By aligning clinical endpoints with stewardship-relevant harms and interpreting pooled effects alongside between-study variability, this work aims to clarify when prophylaxis appears unnecessary, where signals of benefit may exist, and which uncertainties should guide future trials toward standardized, patient-centered, and microbiologically informed outcome reporting.

## Review

Methodology

This systematic review and meta-analysis was conducted according to the PRISMA guidelines. The literature search was carried out in PubMed, Embase, and Cochrane.

Eligibility Criteria

Studies were eligible if they published within the past 10 years in the English language, had a randomized controlled design, and extractable outcome data compared perioperative antimicrobial/antibiotic prophylaxis (ATBP), including preoperative single-dose and perioperative systemic regimens and/or local antiseptic strategies, with placebo and/or no prophylaxis (P/NP) for invasive dental procedures (including implant surgery and extraction-based procedures). Trials were excluded if they lacked an ATBP-versus-comparator contrast, did not report extractable quantitative outcome data, or evaluated noninvasive care without a procedural component. For quantitative synthesis, studies were retained if they reported at least one outcome that could be harmonized across trials, prioritizing early procedure-related infectious outcomes and/or bacteremia-related bloodstream endpoints, with secondary consideration of postoperative morbidity indicators (e.g., pain/recovery measures where available). 

Data Extraction

Three reviewers independently extracted data using Google Sheets (Google LLC, Mountain View, California, United States). Extracted variables included study design, procedure type (implant surgery, extraction, third molar surgery), antibiotic/antimicrobial regimen (agent, dose, timing), comparator (placebo/no prophylaxis), follow-up window for early outcomes, and numeric outcome data (mean, SD, sample size per arm) [21]. 

To maintain consistency with the provided forest plots, the primary endpoint (procedure-related bacteremia incidence or closely related bloodstream bacterial outcomes) was operationalized in Review Manager (RevMan) version 5.4 (2020; The Cochrane Collaboration, London, United Kingdom) by entering event proportions on a 0-1 scale (mean = proportion), enabling computation of a mean difference (MD) aligned with the figure-based dataset. 

For the secondary synthesis, continuous postoperative outcomes reported in a compatible format across a subset of trials were extracted as means and SDs per arm for pooling. 

Where split-mouth designs were used, extraction followed the same analytic structure as the supplied RevMan plots (participant-level when available; otherwise, site-level totals as reported) to avoid introducing post hoc re-derivations that would deviate from the figure-defined dataset. 

Data Analysis

Meta-analysis was conducted in RevMan version 5.4 using an inverse-variance random-effects model (DerSimonian-Laird) to account for expected clinical and methodological variability across procedures, prophylaxis regimens, and outcome ascertainment. Three comparisons were prespecified: (i) invasive dental procedures (all included studies), (ii) a dental surgery subgroup, and (iii) a secondary continuous postoperative outcome in trials with compatible reporting.

Pooled effects were summarized as mean differences (MDs) with 95% confidence intervals (CIs) under the random-effects inverse-variance approach, and statistical significance was set at P < 0.05 (overall Z test). Between-study heterogeneity was assessed using the Cochrane Q (χ²) test and quantified with I².

Risk of bias was evaluated using standard Cochrane domains (selection, performance, detection, attrition, reporting, and other bias), with additional consideration of split-mouth design-specific issues [22-24]. Potential small-study effects/publication bias were explored using funnel plots (standard error versus effect size) for the invasive dental procedure synthesis, interpreted cautiously given the limited number of studies and substantial heterogeneity [25-28].

Results

**Figure 1:**
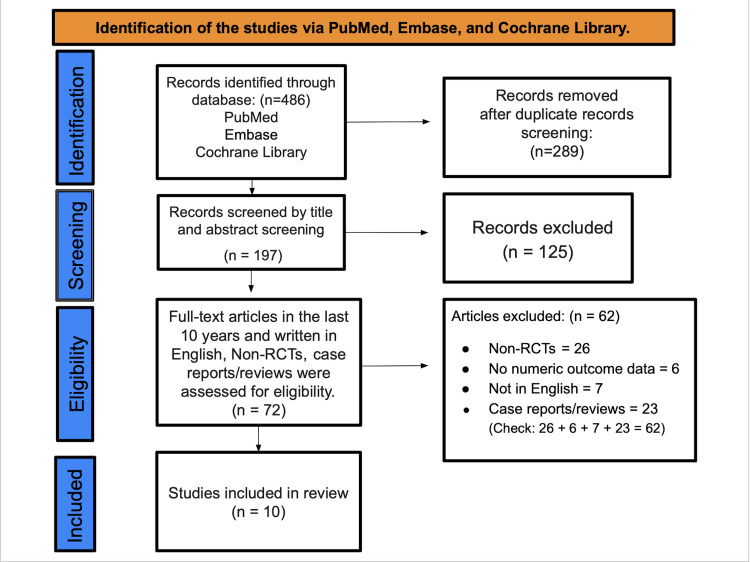
PRISMA flowchart PRISMA: Preferred Reporting Items for Systematic Reviews and Meta-Analyses; RCT: randomized controlled trial

A total of 486 records were identified across PubMed, Embase, and Cochrane. After removal of 289 duplicates, 197 records underwent title/abstract screening, and 125 were excluded. A total of 72 full-text articles were assessed against prespecified criteria, following which 62 were excluded (26 non-RCTs, six without extractable numeric outcomes, seven non-English, and 23 case reports/reviews). Ultimately, 10 studies met all criteria and were included in the qualitative synthesis and meta-analysis (Figure 1). This systematic review and meta-analysis was conducted and reported in accordance with the PRISMA 2020 guidelines [29].

Study Characteristics

In total, the primary meta-analysis (“Invasive Dental Procedure”) pooled 1,950 units of analysis (ATBP=976; comparator=974) across the 10 studies [1-10]. Notably, the unit of analysis was not uniform across the evidence base: depending on study design and procedure type, denominators reflected patients, procedural episodes, or implant/extraction sites, which is a plausible contributor to between-study inconsistency. No study assessed the downstream impact of prophylaxis on rare patient-level outcomes such as infective endocarditis or sepsis; instead, trials focused on early post-procedure endpoints and/or surrogate microbiological outcomes in the immediate perioperative window.

Clinical settings and procedure types varied substantially. Trials encompassed outpatient invasive dental care, including routine extractions, third-molar surgery, and implant placement-procedures with different baseline event rates, bacterial inoculum exposure, and tissue trauma profiles [4-6,8-10]. Follow-up duration also differed across studies (from early postoperative assessments to multi-week monitoring), which can influence the probability of detecting infections or early implant failures and complicate direct comparability [5-10].

Prophylaxis modalities and regimens were heterogeneous, reflecting real-world prescribing variation. Most studies investigated systemic perioperative antibiotics (ATBP), typically as single-dose preoperative regimens or perioperative courses, contrasted with placebo or no antibiotic exposure [6-10]. Several studies evaluated amoxicillin-based prophylaxis (including single preoperative dosing strategies) in implant and extraction contexts [6,8-10]. Alternative regimens were also represented, including clindamycin prophylaxis in implant surgery to address penicillin-allergy pathways and common practice patterns [7]. In addition to systemic antibiotics, at least one trial tested procedural antisepsis (e.g., chlorhexidine mouthwash) as a targeted strategy to reduce procedure-associated bacteremia without systemic antibiotic exposure [1]. One study evaluated an intravenous beta-lactam/beta-lactamase inhibitor approach explicitly framed around bacteremia prevention following dental procedures, representing a higher-intensity prophylaxis paradigm than single-dose oral regimens [2]. This breadth of exposures introduced clinical heterogeneity that is likely to translate into statistical heterogeneity in pooled analyses.

Outcome selection and measurement approaches also differed, representing another major source of inconsistency. Some trials prioritized microbiological endpoints (bacteremia incidence or related surrogate measures) soon after the procedure, whereas others emphasized clinical endpoints (early implant failure, postoperative infections, or patient-reported morbidity) over longer follow-up intervals [1-10]. This is important because prophylaxis may plausibly reduce transient bacteremia without necessarily improving patient-centered clinical outcomes in low-risk settings; conversely, implant failure is multifactorial and may not respond meaningfully to a single antibiotic dose when asepsis, surgical technique, and host factors dominate risk [6-9].

Taken together, these design differences meant that the overall evidence base resembled a portfolio of related, but not interchangeable, clinical questions: (i) can perioperative antimicrobials reduce procedure-associated bacteremia, and (ii) do routine prophylaxis strategies improve early clinical outcomes in implant/extraction care? This conceptual heterogeneity is consistent with the very high statistical heterogeneity observed in the principal pooled comparison and subgroup analysis.

Characteristics of included studies evaluating ATBP in oral surgery and implant-related care are given in Table 1.

**Table 1:** Study characteristics of included trials evaluating peri-procedural antimicrobial/antiseptic prophylaxis for invasive dental procedures and implant surgery (10 studies). Values are n (%) unless otherwise indicated. In split-mouth trials, n refers to the number of surgical sites per condition (participants act as their own control). Amox: amoxicillin; Amox/Clav: amoxicillin–clavulanate; ASA: American Society of Anesthesiologists; CHX: chlorhexidine; CHX-MW: chlorhexidine mouthwash; DB: double-blind; IR: irrigation; IV: intravenous; RCT: randomized controlled trial; SubIR: subgingival irrigation; SupraIR: supragingival irrigation; SSI: surgical site infection

Study (Author, Year)	Design	Procedure/setting	Sample size and allocation, n (%)	Follow-up	Key outcomes reported
Barbosa et al. [1], 2015	Randomized clinical trial (4 parallel arms)	Tooth extraction (bacteremia-focused)	N=201: Control 49 (24.4%); CHX-MW 53 (26.4%); CHX-MW+SubIR 51 (25.4%); CHX-MW+SupraIR 48 (23.9%)	Immediate post-extraction (bacteremia assessment)	Post-extraction bacteremia prevalence and bacterial load (CHX protocols vs. control).
Khalil et al. [2], 2016	Prospective before-after (participants as own control)	Healthy volunteers (single-dose amoxicillin exposure)	N=29 (100%)	Days 1 (baseline), 2, 5, 10, 17, 24	Oral microflora changes and selection for amoxicillin resistance after a single 2 g dose.
Limeres Posse et al. [3], 2016	Randomized controlled trial	Dental procedures (bacteremia-focused)	N=266: Amox/Clav 135 (50.8%); Placebo 131 (49.2%)	Blood cultures at 30 and 60 s post-procedure	Incidence/magnitude of bacteremia; adverse events.
Yves et al. [4], 2021	Randomized multicenter clinical trial	Simple dental extractions	N=142: 48-h regimen 71 (50.0%); 7-day regimen 71 (50.0%)	Day 1 and Day 7 post-extraction	Surgical site infection (primary); dry socket, edema, and pain (secondary); 48-hour vs 7-day amoxicillin.
Kirnbauer et al. [5], 2022	Randomized, placebo-controlled split-mouth trial	Bilateral third-molar removal (within-subject)	N=50 participants; 100 sites: Antibiotic sites 50 (50% of sites) vs placebo sites 50 (50% of sites)	Day 7	Surgical site infection and postoperative sequelae (pain, swelling, and trismus).
Momand et al. [6], 2022	Multicenter double-blind placebo-controlled RCT	Dental implant surgery	N=474: Amoxicillin 238 (50.2%); Placebo 236 (49.8%)	Clinical visits at 7 and 12 days post-surgery	Early implant failure, postoperative infection, and adverse events.
Santamaría Arrieta et al. [7], 2023	Randomized placebo-controlled clinical trial	Dental implant surgery	N=62: Clindamycin 31 (50.0%); Placebo 31 (50.0%)	Days 1, 7, 14, 28, 56	Early implant failure and postoperative complications: preoperative clindamycin (600 mg) vs. placebo.
Momand et al. [8], 2024	Systematic review and meta-analysis	Implant surgery (evidence synthesis)	Included 7 RCTs (n=1859 participants); 17 full-text studies assessed	As reported in included trials (varied)	Early implant failure, postoperative infection, and adverse events with prophylactic antibiotics vs. placebo/no antibiotic.
Bravo-Olmedo et al. [9], 2025	Randomized, blinded, placebo-controlled clinical trial	Dental implant placement	N=120: Amoxicillin 60 (50.0%); Placebo 60 (50.0%)	Days 7, 14, 30, 90	Early implant failure and postoperative infectious complications with/without preoperative amoxicillin (2 g).
García-Blanco et al. [10], 2025	Randomized controlled trial (double-blind)	Single simple dental extractions (type 2 diabetes)	N=56: Amoxicillin 28 (50.0%); Placebo 28 (50.0%)	Day 2 and Day 14 (phone); Day 7 (clinic)	Post-extraction complications (pain, swelling, bleeding, trismus, infection).

Table 2 shows the results of the risk of bias assessment (RoB; Cochrane, London, United Kingdom) for included studies. 

**Table 2:** Cochrane Risk of Bias Assessment Judgments were categorized as Low, High, Some concerns, Unclear, or Not applicable (N/A)

Study (Year)	Random sequence generation	Allocation concealment	Blinding of participants and personnel	Blinding of outcome assessment	Incomplete outcome data	Selective reporting	Other bias	Overall judgment	Notes (concise rationale)
Barbosa et al. [1], 2015	Some concerns	Some concerns	High	Low	Low	Some concerns	Some concerns	Some concerns	Registered trial; intervention blinding not clearly described; analysis reported as blinded.
Khalil et al. [2], 2016	High	High	High	Some concerns	Some concerns	Some concerns	Some concerns	High	Non-randomized before–after design (antibiotic exposure); Cochrane RCT domains not directly applicable.
Limeres Posse et al. [3], 2016	Low	Some concerns	High	Low	Low	Low	Low	Some concerns	Computer-generated randomization; single-blind (microbiologist blinded); allocation concealment procedure not fully detailed.
Yves et al. [4], 2021	Low	Some concerns	High	High	Low	Some concerns	Some concerns	High	Token-draw randomization; allocation handled by examiner; no blinding of patients/clinicians; subjective outcomes possible.
Kirnbauer et al. [5], 2022	Low	Low	Low	Low	Low	Some concerns	Low	Some concerns	Computer-generated blocked randomization; sequentially numbered opaque sealed envelopes; double-blind placebo-controlled split-mouth design; protocol details not fully accessible.
Momand et al. [6], 2022	Low	Low	Low	Low	Low	Low	Low	Low	Computer-generated blocked randomization; code list sealed; placebo-controlled double-blind trial with protocol registration.
Santamaría Arrieta et al. [7], 2023	Low	Low	Low	Low	Low	Low	Low	Low	Independent 1:1 randomization with sequentially numbered opaque sealed envelopes; double-blind placebo-controlled trial.
Momand et al. [8], 2024	N/A	N/A	N/A	N/A	N/A	N/A	N/A	N/A	Systematic review; Cochrane RCT risk-of-bias domains are not applicable.
Bravo-Olmedo et al. [9], 2025	Low	Low	Low	Low	Low	Low	Low	Low	Computer-generated randomization; external keeper of code; identical placebo; blinding reported for operators and analysis.
García-Blanco et al. [10], 2025	Some concerns	Some concerns	Low	Low	Low	Some concerns	Low	Some concerns	Randomized double-blind placebo-controlled trial; randomization/concealment methods not fully described in accessible text.

Evidence Synthesis: Principal Outcomes and Implications for Practice

**Figure 2:**
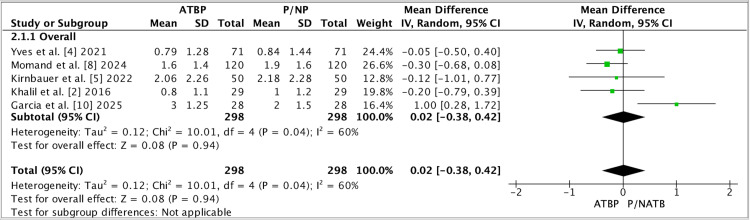
Forest plot (overall analysis). Forest plot comparing antimicrobial/antibiotic prophylaxis (ATBP) versus placebo/no prophylaxis (P/NP) for the prespecified continuous outcome used in this meta-analysis. Individual studies are shown as green squares (point estimate), with square size proportional to inverse-variance weight; horizontal lines represent the 95% confidence interval (CI). The pooled effect is displayed as a black diamond using a random-effects inverse-variance model (IV, Random). The vertical solid line denotes no effect (MD = 0); values to the left favor ATBP, and values to the right favor P/NP, as labeled on the x-axis [2,4,5,8,10].

**Figure 3:**
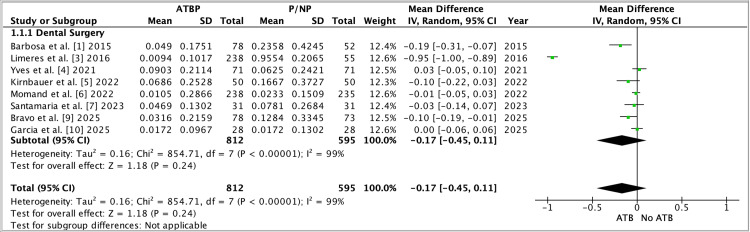
Forest plot (Dental Surgery subgroup label used in RevMan). Forest plot summarizing studies categorized under the single subgroup label “Dental Surgery”, comparing antimicrobial/antibiotic prophylaxis (ATBP) versus placebo/no prophylaxis (P/NP) using mean difference (MD) with a random-effects (IV, Random) model. Study weights, 95% CIs, and the pooled estimate are presented using the same conventions as in Figure F2 [1,3-7,9,10].

Between-study heterogeneity was formally evaluated using Cochrane’s χ² (Q) test and quantified using I², with corresponding χ² p-values reported in each forest plot. Pooled effects were computed in RevMan 5.4 using the inverse-variance random-effects model (DerSimonian-Laird), and the overall treatment effect was tested using the Z test (reported as “Test for overall effect: Z=…, P=…”). Where subgroup analyses were presented (e.g., dental surgery subgroup), RevMan additionally reports a χ² test for subgroup differences with its associated p-value. Notably, these figures summarize study-level pooled comparisons; therefore, participant-level tests such as t-tests were not applied in the meta-analysis itself (any t-tests would only appear within individual trials’ original analyses, not in the pooled RevMan outputs). Figure 2 presents the pooled effect for the secondary continuous outcome using a random-effects model in RevMan 5.4, with heterogeneity reported by χ² and I². Figure 3 shows the dental-surgery subgroup meta-analysis, and the pooled estimate crosses the null with substantial heterogeneity. 

**Figure 4:**
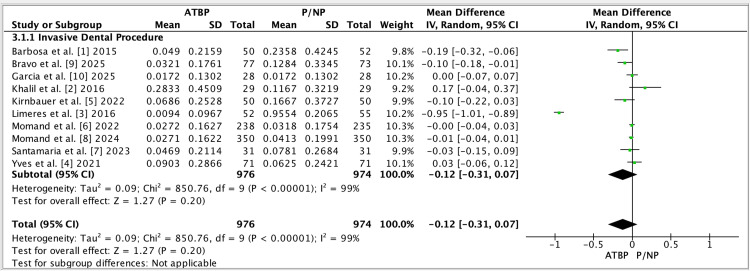
Forest plot (Invasive Dental Procedure subgroup label used in RevMan). Forest plot for the single subgroup label “Invasive Dental Procedure,” pooling all eligible studies comparing antimicrobial/antibiotic prophylaxis (ATBP) versus placebo/no prophylaxis (P/NP). Effect estimates are reported as MD (IV, Random, 95% CI). Between-study heterogeneity is quantified using Tau², Chi², and I², with df indicating degrees of freedom; the overall pooled-effect significance is summarized by the Z test [1-10].

**Figure 5:**
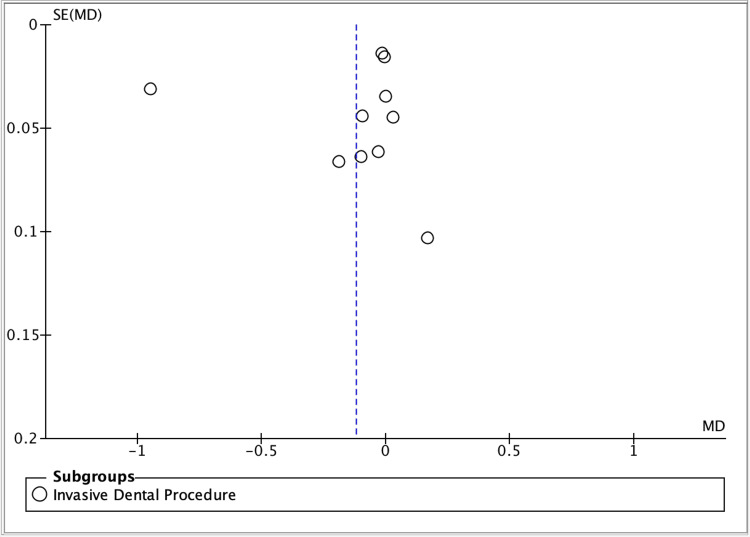
Funnel plot (small-study effects/publication bias assessment). Funnel plot assessing potential small-study effects for the meta-analysis, plotting standard error (SE) of the mean difference (MD) on the y-axis against MD on the x-axis. Each open circle represents an individual study. The vertical dashed line indicates the pooled effect estimate. Visual asymmetry may suggest small-study effects (including, but not limited to, publication bias), particularly when the number of included studies is limited.

Figure 4 summarizes the overall meta-analysis across invasive dental procedures, showing no statistically significant pooled effect and very high between-study heterogeneity. Figure 5 displays the funnel plot used to explore small-study effects, which is interpreted cautiously because heterogeneity is extreme. 

Discussion

Across the included literature, prophylactic antimicrobial or antiseptic regimens were examined largely against placebo or no additional intervention, with endpoints spanning transient bacteremia after oral procedures, postoperative infectious complications following dental extractions, and early implant failure after implant placement [5]. Collectively, the evidence indicates that peri-procedural interventions can modify short-term microbiological outcomes (especially immediate post-procedural bacteremia), whereas effects on downstream clinical outcomes (e.g., surgical site infection (SSI), dry socket, early implant failure) are more inconsistent and likely contingent on baseline risk, procedural complexity, and outcome definitions. This overall pattern is biologically plausible: tissue manipulation and mucosal disruption create a brief window of heightened microbial translocation and contamination, such that interventions timed to that window may exert the largest marginal effect [4-10].

Microbiological Outcomes and Procedural Bacteremia

Studies that evaluated bacteremia shortly after extractions or dental procedures provide mechanistic support for prophylaxis as a means to reduce the initial microbial inoculum entering the bloodstream. In the four-arm RCT by Barbosa et al., chlorhexidine-based prophylactic protocols were associated with differences in post-extraction bacteremia metrics relative to control conditions, reinforcing the concept that reducing oral microbial load and dispersal at the moment of extraction can influence bloodstream detection [1]. Similarly, Khalil et al. examined intravenous amoxicillin-clavulanate administered around dental procedures and measured bacteremia at very early timepoints, again showing that systemic antimicrobial exposure aligned with the procedure can affect bacteremia-related endpoints [2]. While transient bacteremia is not synonymous with clinically meaningful infection, these outcomes remain relevant: they inform biological plausibility, may be important in selected high-risk contexts (e.g., individuals at risk of infective endocarditis), and clarify that “no clinical effect” is not necessarily equivalent to “no antimicrobial effect.”

Clinical Outcomes After Extractions and Oral Surgery

Evidence for clinically meaningful benefits in routine extraction settings is more variable, which may reflect relatively low baseline event rates and heterogeneity in both surgical technique and definitions of postoperative complications [8]. An RCT comparing a 48-hour versus seven-day amoxicillin regimen after simple dental extractions reported that shortened therapy could perform comparably to longer courses for infection-related outcomes, suggesting that prolonged exposure may not translate into proportional additional benefit once the highest-risk early period has passed [4]. Conceptually, this resembles a diminishing-returns curve: the incremental gain in preventing infection falls as early contamination is controlled, while cumulative harms (adverse events, microbiome disruption, and selection pressure) increase with longer exposure. Within oral surgery more broadly, a placebo-controlled split-mouth RCT in routine third molar removal adds further nuance, addressing a common procedure with substantial postoperative morbidity but often low infectious event rates; such designs are informative for evaluating whether prophylaxis meaningfully alters SSI or postoperative sequelae under typical clinical conditions [6]. Taken together, these data support a cautious stance toward routine prophylaxis for uncomplicated procedures in otherwise healthy patients, emphasizing that the absolute risk reduction, if present, may be small.

Implant Dentistry: Early Implant Failure and Postoperative Infection

Implant-related trials are particularly clinically relevant because early implant failure represents a meaningful adverse outcome that may plausibly relate to early microbial colonization and infection [3,6,9,11]. A large multicenter double-blind placebo-controlled RCT evaluated amoxicillin prophylaxis and assessed early implant failure and postoperative infection, providing one of the strongest direct tests of benefit due to its sample size and design rigor [5]. More recent randomized evidence has similarly examined preoperative amoxicillin (e.g., 2 g regimens) compared with placebo and followed participants for early implant failure and postoperative infectious complications [10,14,16]. Across such trials, the direction of effect has not been uniformly large, and the clinical significance likely depends on baseline risk: in low-risk implant placement, event rates are often low, making it difficult for even well-designed studies to demonstrate large absolute risk reductions. A randomized placebo-controlled trial of preoperative clindamycin (often relevant to penicillin-allergic patients) did not provide strong support for universal routine use to reduce early implant failure, raising questions about whether alternative antibiotics offer comparable prophylactic effectiveness or whether their value is restricted to specific indications [7,18,19]. Importantly, a trial focusing on patients with diabetes undergoing extractions introduces the question of subgroup effects; although diabetes may increase susceptibility to infection and delayed healing, “higher risk” does not automatically imply a favorable net benefit, particularly when antibiotic exposure carries its own risks [10]. This highlights the need for future trials to predefine clinically credible subgroups (e.g., poorly controlled diabetes, smokers, grafting procedures, and longer surgeries) and assess effect modification rather than extrapolating from average effects.

Resistance Ecology and the Stewardship Imperative

A central counterbalance to prophylactic use is ecological harm and resistance selection, which can occur even after limited exposure. Khalil et al. demonstrated that a single 2 g amoxicillin dose can measurably alter oral microflora and apply selection pressures relevant to antimicrobial resistance [2]. This finding is critical for interpretation: if the clinical benefit of routine prophylaxis is modest (especially in low-risk procedures), then even small ecological harms may outweigh gains at a population level. Accordingly, prophylaxis decisions should be framed not only in terms of individual-level outcomes but also antimicrobial stewardship principles, which prioritize limiting unnecessary exposure and selecting regimens with the smallest effective spectrum and duration.

Synthesis and Interpretation Across the Evidence Base

The systematic review and meta-analysis by Momand et al. provides an aggregate perspective on implant surgery, synthesizing randomized evidence on early implant failure, postoperative infection, and adverse events [8]. Meta-analytic estimates can stabilize inference when individual trials are underpowered for rare outcomes; however, conclusions remain constrained by clinical and methodological heterogeneity [2,6,8]. Across the included trials, prophylaxis regimens differ in antibiotic choice, timing (preoperative-only vs extended), dosing, and whether postoperative courses were used [13]. Additionally, endpoints such as “infection,” “complication,” and “early implant failure” are not always defined identically, and follow-up windows vary [13]. Such variability introduces heterogeneity that can dilute true effects or create apparent differences unrelated to prophylaxis itself. Another practical limitation is that operator experience, surgical duration, flap design, need for grafting, and adherence to postoperative care are seldom harmonized across studies, yet each can substantially influence infection risk and implant outcomes [17,19].

Implications for Practice and Future Research

Several actionable implications emerge. First, if prophylaxis is selected, evidence supports focusing on timing around the procedure and minimizing duration, since extended courses may not confer additional benefit in straightforward cases while increasing exposure-related harms [4]. Second, routine prophylaxis for all patients and all procedures is difficult to justify given inconsistent clinical outcome benefits and the demonstrable ecological impact of antibiotics [2]. A risk-stratified approach is more defensible, reserving antibiotics for scenarios with higher baseline risk (e.g., complex implant cases, simultaneous augmentation, extensive flap elevation, prolonged surgery, immunocompromise, prior infection history, or other validated risk factors), while relying on strict asepsis, surgical technique, and local antiseptic measures for routine cases [5-8,10]. Third, future RCTs should prioritize standardized outcome definitions (SSI, early implant failure), consistent follow-up windows, and robust adverse-event capture. Incorporating resistance-related endpoints (e.g., carriage of resistant oral flora, microbiome perturbation markers) would directly inform net-benefit assessments, addressing the stewardship gap highlighted by microbiological ecology data [2]. Finally, adequately powered pragmatic trials that compare single-dose prophylaxis versus placebo/no antibiotic, with prespecified subgroup analyses (e.g., diabetes status, smoking, grafting) would help delineate the clinical contexts in which prophylaxis yields meaningful benefit without disproportionate harm [7-10].

In summary, the included studies suggest that peri-procedural antiseptic and antibiotic strategies can influence immediate microbiological endpoints such as bacteremia [1,2], while benefits for preventing postoperative infection or early implant failure appear more modest and variable in routine cases [5-8,10]. Given that even single-dose antibiotic exposure can alter oral microbial ecology and contribute to resistance selection [2], the most evidence-consistent strategy is an indication-driven, risk-adapted approach that minimizes duration and prioritizes stewardship, while future research focuses on harmonized outcomes, clinically relevant subgroups, and direct measurement of ecological trade-offs [4,8].

## Conclusions

The evidence from the 10 included studies suggests that peri-procedural antimicrobial and antiseptic strategies can reduce immediate microbiological endpoints (notably procedure-related bacteremia) compared with no intervention, supporting contamination control during oral surgery. However, for routine extractions and straightforward implant placement, randomized placebo-controlled data indicate that the absolute clinical benefit for preventing postoperative infection or early implant failure is generally modest and inconsistent and may depend on baseline risk and procedural complexity.

Shorter antibiotic courses appear comparable to longer regimens in some extraction settings, favoring exposure minimization when prophylaxis is used. Importantly, even single-dose antibiotic exposure can alter oral microflora and contribute to resistance selection, underscoring antimicrobial stewardship. Overall, a risk-stratified approach, prioritizing minimal effective regimens and reserving antibiotics for higher-risk scenarios, is most consistent with the current evidence base.
